# Impact of the COVID-19 Pandemic on Inappropriate Use of the Emergency Department

**DOI:** 10.3390/microorganisms11020423

**Published:** 2023-02-07

**Authors:** Abelardo Claudio Fernández Chávez, Jesús María Aranaz-Andrés, Miriam Roncal-Redin, Fernando Roldán Moll, María Jesús Estévez Rueda, Patricia Alva García, Yolanda Aranda García, Diego San Jose-Saras, on behalf Health Outcomes Research Group of the Instituto Ramón y Cajal de Investigación Sanitaria (IRYCIS)

**Affiliations:** 1 Servicio de Medicina Preventiva y Salud Pública, Hospital Universitario Ramón y Cajal, IRYCIS, 28034 Madrid, Spain; 2 Facultad de Ciencias de la Salud, Universidad Internacional de La Rioja, 26006 Logroño, Spain; 3 Servicio de Medicina Preventiva y Salud Pública, Hospital Universitario Ramón y Cajal, IRYCIS, CIBER de Epidemiología y Salud Pública (CIBERESP), 28034 Madrid, Spain; 4 Subdirección Médica, Hospital Universitario Ramón y Cajal, 28034 Madrid, Spain; 5 Servicio de Urgencias, Hospital Universitario Ramón y Cajal, 28034 Madrid, Spain; 6 Departamento de Biología de Sistemas, Facultad de Medicina y Ciencias de la Salud, Universidad de Alcalá, 28871 Alcalá de Henares, Spain

**Keywords:** appropriateness, emergency department, COVID-19

## Abstract

**Background**: Inappropriate use of the emergency department (IEDU)—consisting of the unnecessary use of the resource by patients with no clinical need—is one of the leading causes of the loss of efficiency of the health system. Specific contexts modify routine clinical practice and usage patterns. This study aims to analyse the influence of COVID-19 on the IEDU and its causes. **Methods**: A retrospective, cross-sectional study conducted in the emergency department of a high-complexity hospital. The Hospital Emergency Suitability Protocol (HESP) was used to measure the prevalence of IEDU and its causes, comparing three pairs of periods: (1) March 2019 and 2020; (2) June 2019 and 2020; and (3) September 2019 and 2020. A bivariate analysis and multivariate logistic regression models, adjusted for confounding variables, were utilized. **Results**: In total, 822 emergency visits were included (137 per period). A total prevalence of IEDU of 14.1% was found. There was a significant decrease in IEDU in March 2020 (OR: 0.03), with a prevalence of 0.8%. No differences were found in the other periods. A mistrust in primary care was the leading cause of IEDU (65.1%). **Conclusions**: The impact of COVID-19 reduced the frequency of IEDU during the period of more significant population restrictions, with IEDU returning to previous levels in subsequent months. Targeted actions in the field of population education and an improvement in primary care are positioned as strategies that could mitigate its impact.

## 1. Introduction

An increase in the demand for emergency health care is one of the major challenges facing healthcare systems worldwide [1,2]. The inappropriate use of the emergency department (IEDU)—consisting of the unnecessary use of the resource by patients with no clinical need—affects sustainability, efficiency, and quality of care due to unnecessary resource consumption [3] and delays in caring for patients who urgently need assistance [4]. 

An analysis of IEDU is essential to understand the magnitude of the problem. IEDU has traditionally been associated with younger, less clinically severe patients [5,6]. However, the lack of criteria to define what is an inappropriate use has created a great variability in its frequency in the health system [7,8]. It is estimated that between 10 and 90% of emergency care could be inappropriate [9]. To avoid this, several tools have been developed to establish a common definition and comparability across the results. Among these tools, the Hospital Emergency Suitability Protocol (HESP) [10] stands out: it has been validated and is widely used, with an optimal predictive value for detecting IEDU [10]. 

IEDU is not a constant phenomenon in time. It is influenced by the socio-health context, accessibility to the system, and the frequency and patterns of overuse, which change in specific situations [11]. The recent COVID-19 pandemic, which caused a global challenge for health services around the world [12], is expected to have been a factor causing changes in the IEDU, although there have been no studies analysing it. The purpose of this work is to analyse the influence of the COVID-19 pandemic on IEDU in a high-complexity hospital by comparing three pre-pandemic periods with three pandemic periods, adjusted for possible confounders, and studying the causes that produce overuse of the emergency department.

## 2. Materials and Methods

### 2.1. Design, Sample Selection, and Measuring Instruments

A retrospective descriptive study with a cross-sectional analytical design was performed. Three pairs of periods were studied. The first period included 30 March to 5 April, the second was from 15 to 21 June, and the third was from 21 to 27 September, corresponding to the years 2019 (before COVID-19) and 2020 (during COVID-19). The paired periods were: (1) March 2019 and 2020; (2) June 2019 and 2020; and (3) September 2019 and 2020. March 2020 was the period in which the strictest confinement was applied. For June and September 2020, the measures were relaxed, and were mainly limited to the use of masks and crowd control measures. Additionally, during the period of March, there were some primary care centres in the area that could not offer face-to-face care, while usual care was recovered in the months of June and September.

Adult patients over 18 years of age who attended the emergency department of a high-complexity hospital (Hospital Universitario Ramon y Cajal, Madrid) in the mentioned periods were included in the study. In order to obtain enough samples to find significant differences, the score test estimated the sample size with a reference value of 20% (2020) and 30% (2019), a power of 80%, and a confidence level of 95%. The calculated *n* was 137 for each period and year (three periods in 2019 and three periods in 2020), with a total of 822 patients. It is estimated that the hospital emergency department saw 154,607 patients in 2019 and 121,244 patients in 2020. The total number of patients seen for each week of the period was 3415 patients in March 2019 and 2285 in March 2020; 3290 in June 2019 and 2419 in June 2020; and 3049 in September 2019 and 2543 in September 2020.

A random selection was made using STATA 14 [13] applying the runiform command [Syntax: random gen=runiform(); bysort year (random): gen n=_n]. Patients classified in the different areas of the emergency department according to the Manchester scale were selected proportionally, including yellow, orange, and green. Patients classified in the extreme categories of the scale (red and blue) were excluded so that the comparison between groups makes sense, since patients categorized as red will demonstrate appropriate use and blue patients will demonstrate inappropriate use by definition.

The instrument used to identify IEDU was the Hospital Emergency Suitability Protocol (HESP) (Appendix A). The HESP is a tool inspired by the Appropriateness Evaluation Protocol [14]. A panel of experts established a series of criteria that would make emergency care appropriate based on the clinical situation of the patient and the complementary tests carried out. If a visitor does not meet any of the criteria, it is considered inappropriate, and the care could have been managed in an outpatient setting. The tool is based on a retrospective review of the medical record. The HESP has two characteristics that make it appropriate for the objective of the study: (1) it is diagnostic-independent, which makes it possible to analyse patients who attend with different reasons for consultation with the same criteria, and (2) it is highly specific (98%), with a high positive predictive value (96%) for detecting inappropriate use, in addition to having a high kappa index of concordance between observers (0.97). The HESP consists of a form for analysing the causes of IEDU (Appendix B) [10].

Three pairs of reviewers (six in total), divided by period, were used for data collection. The study was conducted in four phases: (1) sample selection and training of participants in the use of the tool; (2) the assignment of a study period to each reviewer and an analysis of the selected sample with the measurement tool; (3) the resolution of complex cases by consensus with the supervisory team; and (4) the analysis and synthesis of results. The data were collected in an online database using Google forms, using information safeguard mechanisms.

### 2.2. Variables

The variable in the analysis was the IEDU and its causes, obtained by means of the HESP. All consultations that met at least one criteria of the form were considered appropriate. According to the HESP, the causes of IEDU were classified into “Patients mistakenly referred by another doctor”, “Patients who come in due to excessive delay in another care establishment”, “Failure in continuous care”, “Ignorance on the part of the patient of the care establishment”, “Greater confidence in the hospital or mistrust of primary care establishment”, and “Convenience and problems of the patient or his/her environment” [10]. The form states that there can be several causes. The information was collected from the patient's medical clinical record.

The epidemiological variables collected were the date of emergency care, age, sex, and priority classification in care according to the Manchester triage system, which classified the degree of prioritisation by colour coding: the “green” patients were seen in two hours, “yellow” in one hour, and “orange” in ten minutes [15]. These data were obtained from the discharge reports recorded in the electronic medical record. 

### 2.3. Statistical Analysis

The prevalence of IEDU was estimated for the total sample and for each period studied. Percentages, central measures (mean and median), dispersion (standard deviation (SD)), and interquartile range (IR) were estimated, and the confidence interval was calculated at 95%. A bivariate analysis was performed for the IEDU and the epidemiological variables for each period in a paired manner (March 2019 versus March 2020; June 2019 versus June 2020; and September 2019 versus September 2020), using hypothesis testing, Chi2 or Fisher’s test in qualitative variables and quantitative–qualitative variables, and the Student’s *t* or Mann Whitney’s U according to the fulfilment of parametric criteria. All hypothesis contrasts were bilateral, with a p-value significance level of less than 0.05 and a confidence level of 95%.

To analyse the influence of the pandemic, three explanatory models of multivariate logistic regression were performed for each of the three pairs of periods, estimating the odds ratio (OR) of IEDU over one year with respect to the previous year adjusting for confounding factors (age, sex, and priority level on the Manchester scale).

The STATA Statistical software, version 16 (StataCorp. 2019. College Station, TX, USA: StataCorp LLC) was used for statistical analysis [13]. 

### 2.4. Ethics Committee

The study was approved by the Ethics Committee of the Hospital Universitario Ramón y Cajal (10 March 2021, ACT 410).

## 3. Results

### 3.1. Sample Characteristics

**Figure 1:**
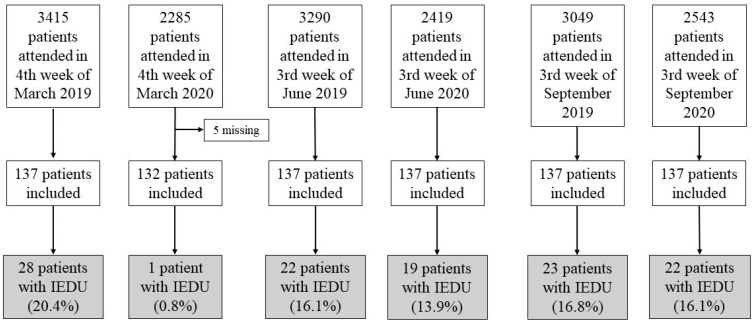
Flow diagram. IEDU: Inappropriate use of the emergency department.

We included 822 patients who attended the emergency room during the months of March, June, and September in 2019 and 2020. Information was collected from 137 emergency services for each month and year. In March 2020, five patients (3.7%) were lost (Figure 1).

Of the 817 emergency services analysed, 115 were inappropriate, representing 14.1% of the total sample. The mean and median ages were 61.6 (SD: 21.2) and 63 (IR: 45 to 80) years, respectively. Of the 817 patients studied, 415 (50.2%) were women and 402 (49.2%) were men. 

A total of 414 patients (50.7%) were classified in the “green” category of the Manchester scale, 275 (33.7%) as “yellow”, and 128 (15.7%) as “orange”. The highest prevalence of IEDU found overall was in patients classified as “green” (26.1% versus 2.2% in yellow and 0.8% in orange; *p* < 0.001).

### 3.2. Prevalence of Inappropriateness by Period and Bivariate Analysis

By means of a stratified analysis of each period, in March 2019, a prevalence of 20.4% of IEDU was found, compared to 0.9% in March 2020 (*p* < 0.001). No differences were found between June 2019 and 2020 (16.1% prevalence versus 13.9%; *p* = 0.611) and between September 2019 and 2020 (16.8% versus 16.1%; *p* = 0.870).

With the exception of March 2020, the increase in the Manchester Scale score resulted in a lower prevalence of IEDU. By periods, the highest prevalence on the Manchester scale was found in March 2019 (a 38.4% prevalence of “greens”; *p* ≤ 0.001), followed in descending order by September 2019 (34.5%; *p* < 0.001), September 2020 (28.0%; *p* < 0.001), June 2020 (25.3%; *p* < 0.001), and June 2019 (25.0%; *p* = 0.003).

Age was associated with IEDU in March 2019 (a median age of 45 years in patients with inappropriate emergency care versus 65 years in the sample for that period; *p* = 0.003), in September 2020 (a median age of 47 years versus 57 years; *p* = 0.027) and in September 2019 (a median age of 50 years versus 63 years; *p* = 0.032) (Table 1).

### 3.3. Multivariate Analysis

After adjusting the multivariate model for age, sex, and classification on the Manchester scale, patients who attended the emergency room in March 2020 had significantly lower IEDU (Odds Ratio (OR) [95% CI]: 0.03 [0.0 to 0.2] versus 2019). No statistically significant differences were found for the June OR [95% CI]: 0.90 [0.4 to 1.8] or September 2020 periods OR [95% CI]: 0.69 [0.3 to 1.4].

In the remainder of the model for each period, an increased severity on the Manchester scale reduced the association with the IEDU in the March periods (OR [95% CI]: 0.04 [0.0 to 0.3] in patients classified as yellow versus patients classified as green in March), June (OR [95% CI]: 0.06 [0.2 to 0.3] in yellow versus green), and September (OR [95% CI]: 0.07 [0.0 to 0.3] in yellow versus green). There were only IEDU in patients classified as orange in September, and there was also a lower association with respect to “greens” (OR [95% CI]: 0.05 [0.0 to 0.4]). (Table 2).

### 3.4. Causes of Inappropriateness

The main cause of IEDU identified was a greater degree of trust in the hospital or a mistrust of a primary care establishment, accounting for 65.1% of the total inappropriate care. This was followed by 12.8% due to an excessive delay in another care establishment and 9.4% due to referral by another doctor.

Stratified by year and period, it was observed that in September 2020, 87.0% of IEDU were due to a greater degree of trust in the hospital, compared to 56.3% in 2019. In September 2019, 40.6% of IEDU were due to an excessive delay in another care establishment, compared to 4.4% in 2020 in the same month. In June 2020 and 2019, 64.3% of IEDU were due to a greater degree of trust in the hospital, followed by 10.7% due to failure to continue care. In March 2019, 79.4% of the IEDU was due to a greater degree of trust in the hospital. The only case of inappropriateness seen in March 2020 was due to a referral by another doctor. 

## 4. Discussion

The results of the study indicate a higher number of inappropriate visits to the Emergency Department of the Hospital Ramón y Cajal in the periods studied in the year 2019 than in 2020. In 2020, March saw the lowest percentage of inappropriateness, coinciding with the imposition of the State of Alarm for COVID-19 in Spain. 

Analysing the IEDU and understanding its frequency is essential for mitigating the problem of inappropriate emergency department use, as an increase in inappropriate use has been associated with an increase in mortality derived from delayed treatment and a higher financial cost [16]. A recent systematic review found a prevalence among the included studies of between 24 and 40%, slightly higher than what was found in our study [17]. In Spain, Aranaz et al. found a prevalence of 30.7% using the HESP in 2004 [5]. Other studies using the same tool also found a higher prevalence of IEDU, although there is a lack of studies with recent measurements [18].

No work identified a reduced prevalence of IEDU such as the one found in March 2020. This moment coincides with the start of the COVID-19 pandemic and the decree of the state of alarm in Spain, after confinement measures and population mobility restrictions were adopted [19]. The pandemic changed usual clinical practice with disparate effects. On one hand, it prompted the overuse of health services in certain tests and treatments [20]. On the other hand, it also caused underuse due to difficult access and fear among the population [21]. Our results suggest that the pandemic decreased the overuse of the department, but it is plausible that this was due to an increase in underuse, as happens in other adverse contexts, such as in regions with higher levels of poverty, inequality, or war, where the reduction of unnecessary practices is secondary to a decrease in accessibility [22,23,24,25].

No differences were observed in the June 2019 and 2020 and September 2019 and 2020 periods. In these months, although certain measures were in place to mitigate COVID-19, regular clinical activity gradually normalised, explaining the lack of difference. Some studies, such as a study by Zaboli et al. in 2022 in Italy, found a patient profile that went to the emergency department with less severity throughout 2021 when compared to before the pandemic [26]. Future studies should analyse whether, after the initial impact in 2020, an opposite phenomenon of overuse of the emergency department occurred later. Be that as it may, these variations suggest that the COVID-19 crisis is a good phenomenon to prompt the reconsideration of the suitability of certain clinical practices and to efficiently reorganise resources to mitigate the impact of IEDU [27].

IEDU has been associated with younger patients in certain periods, such as March 2019 and September 2019 and 2020, remaining somewhat consistent with previous evidence. The HESP, due to its appropriateness criteria, may encourage this connection because the need for tests, which are more likely in older patients, make the visit appropriate. However, the association has been seen in numerous previous studies, regardless of the tool used. Studies of general samples of patients also found an association between IEDU and younger patients [5,28]. On the other hand, in studies that included only patients older than 65 years, a lower prevalence was found: 13.1% [29] and 6.0% [30]. Other studies, focused on appropriate visit factors, found an direct association with increasing age [6]. 

In the relationship between inappropriateness and sex, no association was found in any period, and the prevalence of IEDU remains similar in men and women. In studies more focused on epidemiological analysis, such as a study by Carret et al. in Brazil in 2007, a sex association was found to exist in persons under 50 years of age, with a higher prevalence in women—although this association later disappears with age [31]. This type of association was also found in other studies, including those that used HESP [5] and in those that did not [28]. In our study, the objective was not to analyse the behaviour of sex with respect to age. Based on previous evidence, it was decided to leave sex in the multivariate explanatory models.

Another finding common to all study periods is the association between IEDU and triage severity, with the risk of inappropriateness being more than fourteen times higher in patients triaged as green with respect to yellow. This is constant in the whole sample, and patients classified as less severe on the Manchester scale and younger patients were the patient type most associated with IEDU. In earlier studies, the association of the IEDU with lower patient severity was already known [5,6], although there has been a disparity of criteria for analysing this variable. The study by Zúñiga et al. in 2022, Switzerland, which analysed a sample of patients over 65 years of age, adjusting for triage severity on a scale similar to the Manchester, found an association between inappropriate demand and lower patient severity [30], something common to other forms of overuse, such as inappropriate admission [32,33]. Intermediate values were chosen regarding severity in the Manchester classification (green, yellow, and orange). This is due to the fact that patients categorized as blue almost certainly imply inappropriate care [15], while those categorized as red require immediate care, so inappropriateness is very low in this group. By including only the intermediate categories, a more detailed and focused analysis has been made. Attendance at the emergency department can also influence these results. While the proportion of triage patients is similar in all periods (50–55% in green, 30–40% in yellow and 5–15% in orange), we found that in March 2020, there was an increase in the proportion of oranges (31.8%). This change partly explains this decrease in the inappropriateness produced at that time. This change in the patient profile in the emergency department was also identified in the United States, where they found a decrease in attendance and a more severe patient profile [34].

The study identifies user mistrust of care as the main cause of IEDU. The same main cause has also been identified in numerous studies that analysed the causes of IEDU before the pandemic, including a lack of availability of care in general medicine, the difficulty in making an appointment, and the saturation of primary care [18,31,35]. 

Delays in patient care and an increased mistrust in primary care were identified as the main causes of IEDU; these causes were similar to pre-pandemic periods. The influence of the problems derived in primary care in the context of crisis, both in terms of lack of health personnel or due to an excessive demand for care, and their impact on emergency care, has already been studied in other contexts [36]. The improvement in primary care is positioned as a fundamental measure to reduce IEDU [1]. 

Moreover, most patients who attend inappropriately do so independently, and more than half do so without being aware that they are making inappropriate use of the service [37] making population education a necessary tool to mitigate the impact [38]. 

### Limitations and Strengths

The study has some limitations. First, the retrospective, cross-sectional design allowed us to discuss the association of different periods and variables with inappropriateness, but longitudinal studies are necessary to deepen this analysis and establish causal relationships with a larger collection of epidemiological variables linked to health care. However, this does not prevent this study from revealing the differences in the different periods, and it can be positioned as a possible starting point for future research to complete it.

On the other hand, the HESP tool is based on a review of the electronic medical record and has a subjective component, which can be affected by the quality of data collection in the records and by the interpretation of the reviewer. The HESP, especially in the collection sheets of the causes of IEDU, assumes this possible loss of information in the section of Appendix B, offering some possibilities of causes of inappropriateness that can be reviewed in the clinical history (such as the prior referral from primary care). However, the inappropriateness is difficult to quantify, and HESP is a previously validated and widely recognised tool, which confers greater external validity to the results than other techniques used, such as expert consensus.

Likewise, this study is the first to evaluate the impact of the pandemic caused by COVID-19 on IEDU, providing, in addition, an updated data on the prevalence of IEDU. For this purpose, a representative sample of the hospital population in different pandemic periods and phases has been selected and adjusted for the severity of the patients, age, and sex. The design of the study, comparing the same periods of different years with each other, allows us to evaluate the effect of the confinement measures on the overuse of the emergency department, finding the differences precisely in March 2020, the period with the highest degree of restrictions, and not in June and September 2020, the periods of fewer restrictions. 

In addition, thanks to these results, crucial epidemiological information has been obtained on the profiles of patients who attend the emergency room inappropriately. These groups are ideal target groups for developing mitigation strategies, similar to the way that strategies are developed for other diseases in specific groups according to the risk of complications, such as acute coronary syndrome.

Finally, a validated and widely recognised questionnaire was used as a measurement tool, which confers reliability and comparability to our results.

## 5. Conclusions

The COVID-19 pandemic caused a significant decrease in IEDU during the implementation phase of population-based disease control measures in March 2020, returning to levels similar to previous levels in subsequent months. The decrease that occurred was similar to decreases documented in other crises, and it is plausible that it would lead to an increase in derived underuse. 

Delays in patient care and an increased mistrust in primary care were identified as the main causes of IEDU. These causes are similar to causes in pre-pandemic periods. Younger patients and patients classified as less severe on the Manchester scale were the patient types most associated with IEDU. 

Public education actions targeting this patient profile, coupled with improvements in primary care services, are still positioned as strategies to mitigate the impact of overuse of the emergency department.

## Figures and Tables

**Table 1:** Descriptive by study period and bivariate analysis.

		Total	Total 2019	Prevalence IEDU 2019			Total 2020	Prevalence IEDU 2020		
		n (%)	n (%)	n	% (CI95%)	*p*-Value	n (%)	n	% (CI95%)	*p*-Value
**March**	**Age**									
	Medium (SD)	62.4 (21.1)	61.3 (22.7)	49.6 (22.1)	41.0 to 58.2	0.003 *	63.4 (19.2)	72	−	0.773
	Median (RI)	63 (46 to 81)	65 (41 to 81)	45 (31 to 72)	−		61 (49 to 81)	72	−	
	**Sex**									
	Female	136 (50.6)	71 (51.8)	17	23.9 (14.6 to 35.5)	0.291	65 (49.2)	0	−	1.000
	Male	133 (49.4)	66 (48.2)	11	16.7 (8.6 to 28.9)		67 (50.8)	1	1.5 (0.0 to 8.2)	
	**Manchester**									
	Green	126 (46.8)	73 (53.3)	28	38.4 (27.2 to 50.5)	<0.001 **	53 (40.2)	0	−	0.280
	Yellow	79 (29.4)	42 (30.7)	0	−		37 (28.0)	1	2.7 (0.0 to 14.2)	
	Orange	64 (23.8)	22 (16.19)	0	−		42 (31.8)	0	−	
	**Total**	**269 (100.0)**	**137 (50.9)**	**28**	**20.4 (14.0 to 28.2)**		**132 (49.1)**	**1**	**0.8 (0.0 to 4.1)**	<0.001 **
**June**	**Age**									
	Medium (SD)	61.9 (20.7)	60.9 (21.0)	54.9 (18.4)	46.7 to 63.1	0.119	62.8 (20.3)	56.5 (23.7)	45.1 to 67.9	0.212
	Median (RI)	65 (46 to 79)	62 (45 to 78)	53 (40 to 69)	−		68 (50 to 79)	62 (33 to 76)	−	
	**Sex**									
	Female	140 (51.1)	68 (49.6)	10	14.7 (7.3 to 25.4)	0.669	72 (52.6)	11	15.3 (7.9 to 25.7)	0.805
	Male	134 (48.9)	69 (50.4)	12	17.4 (9.3 to 28.4)		65 (47.5)	8	12.3 (5.5 to 22.8)	
	**Manchester**									
	Green	155 (56.6)	80 (58.4)	20	25.0 (16.0 to 35.9)	0.003 *	75 (54.7)	19	25.3 (16.0 to 36.7)	<0.001 **
	Yellow	100 (36.5)	46 (33.6)	2	4.4 (0.5 to 14.8)		54 (39.4)	0	−	
	Orange	19 (6.9)	11 (8.0)	0	−		8 (5.8)	0	−	
	**Total**	**274 (100.0)**	**137 (50.0)**	**22**	**16.1 (10.3 to 23.3)**		**137 (50.0)**	**19**	**13.9 (8.6 to 20.8)**	**0.611**
**September**	**Age**									
	Medium (SD)	60.6 (21.8)	62.5 (22.0)	53.7 (20.3)	44.9 to 62.5	0.032 *	58.7 (21.6)	49.8 (18.9)	41.4 to 58.2	0.027 *
	Median (RI)	60 (44 to 80)	63 (44 to 83)	50 (39 to 72)			57 (42 to 77)	47 (37 to 65)		
	**Sex**									
	Female	139 (50.7)	68 (49.6)	14	20.6 (11.7 to 32.1)	0.237	71 (51.8)	9	12.7 (5.9 to 22.7)	0.352
	Man	135 (49.3)	69 (50.4)	9	13.0 (6.1 to 23.3)		66 (48.2)	13	19.7 (10.9 to 31.3)	
	**Manchester**									
	Green	133 (48.5)	58 (42.3)	20	34.5 (22.5 to 48.1)	<0.001 **	75 (54.7)	21	28.0 (18.2 to 39.6)	<0.001 **
	Yellow	96 (35.0)	53 (38.7)	2	3.8 (0.5 to 13.0)		43 (31.4)	1	2.3 (0.0 to 12.3)	
	Orange	45 (16.4)	26 (20.0)	1	3.9 (0.0 to 19.6)		19 (13.9)	0	−	
	**Total**	**274 (100.0)**	**137 (50.0)**	**23**	**16.8 (10.9 to 24.1)**		**137 (50.0)**	**22**	**16.1 (10.3 to 23.3)**	**0.870**

IEDU: Inappropriate use of the emergency department; CI95%: 95% confidence interval; * *p* < 0.05; ** *p* < 0.001. *p*-value for percentage differences: Using Chi-square tests (if parametric test conditions are met) and Fisher’s exact test (non-parametric); for median differences: using the Mann-Whitney U test (non-parametric).

**Table 2:** Multivariate analysis of association between inappropriate cases by study period.

		OR	CI 95%		*p*-Value
**March**	**Year (1)**	0.03	0.00	0.23	<0.001 **
	**Age (2)**	0.99	0.97	1.01	0.350
	**Gender (3)**	1.34	0.52	3.43	0.540
	**Manchester (4)**				
	Yellow	0.04	0.01	0.34	0.004 *
	Orange	−	−	−	−
	**Constant**	0.87	0.26	2.92	0.830
**June**	**Year (1)**	0.90	0.44	1.84	0.779
	**Age (2)**	0.99	0.98	1.01	0.547
	**Gender (3)**	1.00	0.50	2.05	0.980
	**Manchester (4)**				
	Yellow	0.06	0.2	0.28	<0.001 **
	Orange	−	−	−	−
	**Constant**	0.47	0.14	1.51	0.200
**September**	**Year (1)**	0.67	0.33	1.37	0.280
	**Age (2)**	0.99	0.98	1.01	0.540
	**Gender (3)**	1.29	0.64	2.61	0.470
	**Manchester (4)**				
	Yellow	0.07	0.02	0.26	<0.001 **
	Orange	0.05	0.00	0.37	0.004 *
	**Constant**	0.64	0.22	1.90	0.430

OR: Odds Ratio; CI95%: 95% Confidence Interval; * *p* < 0.05; ** *p* < 0.001; (1) Year: 2019 = 0; 2020 = 1; (2) Age: risk variation for each increase in one year (3) sex; female = 0, male = 1; (4) Manchester: green = 0, yellow = 1, orange = 2. ** Statistically significant.

## Data Availability

The database can be made available upon request to the authors

## Appendix A

**HESP Scale**	
	**Severity Criteria**
1.1	Loss of consciousness, disorientation, coma, numbness (sudden or very recent)
1.2	Sudden loss of vision or hearing
1.3	Abnormal heartbeat (<50/>140 beats/minute) and arrhythmia.
1.4	Blood pressure disorders (systolic: <90/>200 mmHg; Diastolic: <60/>120 mmHg).
1.5	Electrolyte or blood gas imbalances (Do not consider in patients with chronic imbalances of these parameters: chronic renal failure, chronic respiratory failure, etc.)
1.6	Prolonged fever (5 days) not controlled with treatment in primary care setting.
1.7	Active bleeding (haematemesis, epistaxis, manes, etc.). Excludes superficial wounds that only require suture.
1.8	Sudden loss of functional capacity of any part of the body
	**Treatment Criteria**
2.1	Intravenous medication or fluid administration (except line maintenance)
2.2	Oxygen therapy
2.3	Plaster cast (excludes bandages)
2.4	Surgery/procedure performed in the operating room.
	**Diagnostic INTENSITY CRITERIA:**
3.1	Monitoring vital signs or taking signs every two hours.
3.2	Radiology of any kind.
3.3	Laboratory tests (except blood glucose in diabetics who come for reasons unrelated to diabetes and dry strip blood glucose tests).
3.4	Electrocardiogram (except chronic heart disease who come with problems unrelated to heart disease).
	**Other Criteria**
4.1	The patient is under observation in HED for more than 12 h
4.2	The patient is admitted to the hospital or transferred to another hospital
4.3	The patient dies in the HED
4.9	Other patient referred by doctor (specify).
	**Criteria Applicable Only To Patients Who Come Spontaneously**
5.1	Patient comes in after an accident (traffic, work, in public place,) and needs assessment.
5.2	They appear to be life-threatening emergencies: chest pain, rapid onset dyspnoea, intercostal retraction, acute abdominal pain.
5.3	Condition known to the patient that usually requires admission.
5.4	A doctor has told the patient to go to the emergency room if the symptom occurs
5.5	It requires primary medical care quickly and the hospital is the nearest centre
5.9	Others in spontaneous patients (specify).
HED: Hospital emergency department.	

## Appendix B

**Causes Of Inappropriate Use of Hospital Emergencies**	
1	Patients referred by a doctor
1.1	Not an emergency. Does not require immediate attention
1.2	The patient requires immediate care, which could be provided outside the hospital
1.3	Referred from outpatients to speed up diagnosis
1.4	Forwarded by error
1.9	Other: specify
	Spontaneous patients
2	Excessive delay in another care establishment
2.1	Surgical waiting list
2.2	Hospital outpatient waiting list
2.3	Hospital outpatient consultation (delay between visits)
2.4	Specialist consultation area
2.5	Primary care consultation (by appointment)
2.6	Diagnostic tests requested by primary care or area specialist
2.7	Diagnostic tests requested by the hospital
2.9	Other: specify
3	Failure in ongoing care
3.1	The general practitioner’s consultation has ended
3.2	Delay in primary care home visits
3.3	Delay in emergency service home visits
3.4	Impossible to contact the Health Centre
3.5	Impossible to contact the Emergency Department
3.9	Other: specify
4	The patient does not know how to use the care establishment
4.1	The patient has not been assigned a primary care doctor
4.2	The patient does not know the primary care doctor’s address/telephone number
4.3	The patient does not know the existence\location\phone of the Emergency Department
4.9	Other: specify
5	Increased confidence in the hospital or mistrust of a Primary Care establishment
5.1	Patient has seen the primary care doctor and does not trust them
5.2	Patient has gone to the emergency department and does not trust them
5.3	Patient went directly to the hospital emergency room
5.4	The patient has a history at the hospital and believes they will receive better care
5.9	Other: specify
6	Convenience and problems with the patient or their surroundings
6.1	Lives nearby/not able to get time off work/quick service/more convenient
6.2	Patient problems: Low IQ, hypochondriac, fakes illness
6.3	Wants examination (radiology, analytics)
6.4	The family wants to admit the patient
6.5	On the orders of a public authority: Police, Judge
6.9	Other: specify
